# Exploring the effectiveness of family-based interventions for psychosis in low- and middle-income countries: a systematic review

**DOI:** 10.1007/s00127-022-02309-8

**Published:** 2022-06-14

**Authors:** Hannah Morillo, Sophie Lowry, Claire Henderson

**Affiliations:** 1 King’s College London, London, UK; 2 London School of Hygiene and Tropical Medicine, London, UK

**Keywords:** Psychosis, Family-based interventions, Low- and middle-income countries, Complex mental health interventions, Community mental health

## Abstract

**Purpose:**

Of the 80% people with psychosis living in low- and middle-income countries (LMICs), up to 90% are left to the care of families. The World Health Organization has recommended the inclusion of families in community-based rehabilitation and while there is evidence of its implementation in LMICs, this has not been reviewed yet. This study aims to describe the key features and implementation strategies of family-based interventions in LMICs, and appraise their effectiveness.

**Methods:**

Included are people with psychosis in LMICs who receive any form of family-based intervention, compared to their usual or absence of treatment, with patient outcome measures. We searched (August 2021) through Embase, MEDLINE, Global Health, PsycInfo, Social Policy and Practice, and Cumulative Index to Nursing and Allied Health Literature (CINAHL), as well as from grey literature and hand-searched records. Risk of bias was assessed through the Integrated Quality Criteria for Review of Multiple Study Designs (ICROMS) and Consolidated Health Economic Evaluation Reporting Standards (CHEERS), then analyzed narratively.

**Results:**

27 studies were included from the 5254 records. Psychotherapeutic features, systems approach and task-sharing were key intervention elements. Delivery strategies included preliminary research, sustained family engagement, and cultural adaptation. There were positive health impacts across four outcome domains.

**Conclusion:**

All studies recommended family-based interventions, with limitations in heterogeneity and 70% of them rated high risk of bias.

**Other:**

Review was registered in PROSPERO (CRD42021256856). The authors did not receive funding for this research.

**Supplementary Information:**

The online version contains supplementary material available at 10.1007/s00127-022-02309-8.

## Introduction

Despite the growing evidence on the global burden of psychosis [1–4], there is still an urgent need to scale up services to support people with psychosis and their families in low- and middle-income countries (LMICs) [5]. Pharmacological treatment may be the cornerstone treatment for its practicality in administration, but this is challenged by low adherence and side effects [6]. Social determinants may significantly impact the prevalence of psychosis [7]; a biopsychosocial approach is therefore needed. Apart from this, the availability of second-generation antipsychotics, and in some cases any medication, is variable [8]. Among psychological interventions, cognitive behavioral therapy (CBT) shows encouraging evidence in some contexts [9, 10], but negative symptoms may not be effectively addressed [11]. The World Health Organization (WHO), through the Mental Health Gap Action Plan (mhGAP), has endorsed community-based rehabilitation especially for rural areas in LMICs [8]. However, the challenge for low-resource settings is to provide access to this service, which is made more difficult due to a limited number of mental health specialists [9].

Of the almost 80% of people with psychosis living in LMICs, 90% are primarily cared for by their families [8, 12–16]. Recently, there has been strong emphasis on engaging the family in advancing global mental health interventions [5, 17, 18] and specifically for early onset psychosis [19]. To reinforce community-based rehabilitation, it makes sense to draw upon social capital by task-sharing to family carers, who may be the people working closest with the person with psychosis. Family-based interventions essentially tap the family member(s) of a person with psychosis to be the main delivery agent of care, whether through psychoeducation, family counselling/therapy, or through a combined program with pharmacological treatment [20, 21]. Clinically, when psychoeducation is given in family therapy, negative aspects of expressed emotion (EE), or more specifically, the critical, hostile and emotional over-involvement of the family environment, was effectively reduced and caring for the person with psychosis was improved [22]. Evidence over the past decade has recorded favorable outcomes for family intervention, particularly clinical outcomes, medication compliance, social functioning, family outcomes, and quality of life [23–25]. Additionally, economic analyses point to agreeable outcomes in net household savings and cost-effectiveness [26, 27].

Across cultures, various efforts in implementation have illustrated the feasibility and effectiveness of family-based intervention [20, 25, 28–35]. In high-income countries, there have been challenges in bringing family-based intervention to routine care, but it has already been widely incorporated into mental health services [36]. In scaling up community-based programs, psychoeducation in India [32, 37], Pakistan [38, 39], Ethiopia [40], China [41], and other low-resource countries [42] have benefitted in utilizing key family members. Robust evidence in HICs supports family-based interventions and is being implemented in mental health care facilities. Growing evidence provides a basis for optimism for the uptake of family-based intervention in mental healthcare interventions in LMICs, but a synthesis of delivered family-based interventions and their effectiveness has not yet been conducted. Focusing on the studies in LMICs provides a substantial contribution to the literature of psychosis interventions.

This systematic review aims to (i) describe FBI for psychosis studied in LMICs, which synthesizes intervention features and delivery strategies, and (ii) appraise the evidence of family-based interventions in LMICs. Exploring intervention elements and its delivery could help identify active ingredients in family-based interventions to enable its incorporation within the LMIC context. The output of this systematic review will inform communities in mental health research, clinical practice as well as non-practitioners involved in psychosis interventions and policies.

## Methods

Protocol for this review was approved by the London School of Hygiene and Tropical Medicine Ethics Committee and registered to PROSPERO (CRD42021256856), following the Preferred Reporting Items for Systematic Reviews and Meta-Analyses (PRISMA) reporting guidelines and completed the PRISMA 2020 checklist [43]. A stepwise process of screening the titles and abstracts, then the full-text articles according to the eligibility criteria was performed.

### Eligibility criteria

The study population is people with psychosis in low- and middle-income countries, who received any form of family-based intervention compared to their usual, or absence of, treatment. Broad direct and indirect patient outcomes were included, i.e., from clinical outcomes to a shift in the behavior or the attitude of the family that in turn affects patient outcomes. Since the study aims to explore the existing evidence on family-based intervention in LMICs, it sought to include all relevant studies that reported outcomes related to the people with psychosis. Therefore, cost-effectiveness outcomes were also included. This review was not limited to the year of publication nor a specific time frame, and all study designs were included.

Eligibility criteria were assigned to the following domains: (A) family-based interventions, (B) people with psychosis, and (C) low- and middle-income countries. Family-based intervention is defined as any intervention that involves one or more family member(s) as recipients of the service and agents of its effectiveness on the patient. It includes, but not limited to, family intervention, family therapy, family psychoeducation, family workshops, “crisis intervention support for the family” [44], and “family-focused intervention” [23]. Family therapy, a type of group psychotherapy, is defined as treatment of more than one family member in the same session [45]. Psychoeducation in this review denotes the structured learning of the patient and their family about psychosis and how to manage this within their lives. Within the framework of psychotherapy, its content ranges from the nature of the mental illness, managing symptoms, effective caregiving for the person with psychosis, problem-solving, and treatment modalities. Psychosis is a mental condition characterized by cognitive impairment, disorganized behavior, and a disconnect from reality, including hallucinations and delusions as experiences of positive symptoms and marked unresponsiveness as negative symptoms [46, 47]. Diagnoses were done by mental health practitioners and researchers. In this review, we define a person with psychosis as someone who has been diagnosed with the aforementioned symptoms at any age after onset with early onset, acute or chronic psychosis. The medical subject headings (MeSH) term used for this review is “psychotic disorders”, including schizophrenia and bipolar disorder as they are the most common types. Psychosis due to substance abuse was excluded. Lastly, low- and middle-income countries were defined from the World Bank income division [48], cross-validated with the Organization for Economic Cooperation and Development (OECD) and the LMIC Filters of the Cochrane Database of Systematic Reviews [49]. All the 109 country names and the derivative terms for LMICs, both former and recently used, were included in the search.

### Information sources and search strategy

Studies were searched through the following bibliographic databases: Embase, MEDLINE, Global Health, PsycInfo, Social Policy and Practice, and Cumulative Index to Nursing and Allied Health Literature (CINAHL), through Ovid platform. Scopus and Google Scholar were used to unpublished reports. Relevant Chinese conference proceedings and records that were unavailable were hand-searched from Wangfang Data, a Chinese bibliographic database, ResearchGate, a social networking site for researchers, and through the British Library collection. The electronic search strategy covered the three domains to accommodate the specific database syntax. Consistent with the population, intervention, comparator, outcomes (PICO) approach adopted, search terms were: (A) psychosis (e.g., psychos?s or brief reactive psychos?s or bipolar disorder* or schizoaffective disorder* or schizophren*), (B) family-based interventions (e.g., family-based intervention* OR family therap* OR family-based OR parent* OR mother* OR father* OR primary care-giver* OR sibling*), and (C) low- and middle-income countries (e.g., yemen OR yugoslavia OR zambia OR zimbabwe OR global south OR sub-saharan africa OR lmic OR lmics OR third world OR lami countr*). All terms were combined by the Boolean term “OR” within the domains and “AND” when key terms per domain were combined.

### Selection process

All authors participated in the two-tier screening process for eligibility for preliminary (title and abstract) and full-text articles. HM screened all articles on both stages and SL co-screened all titles and abstracts and 30% of the full-text articles, above the recommended 20% cut-off [50]. Agreement between raters was at least 97.6% inter-rater agreement with Kappa = 0.78. Four discordant articles were resolved by referencing the eligibility criteria and consultation with CH. HM performed an update of the search one year after and CH co-reviewed the included full-text articles, where full agreement of additional included articles was made. Eligibility assessment was performed for all included studies on an individual blinded standardized manner via Rayyan (https://rayyan.ai/), a free web-based collaboration and reviewing tool.

### Data collection process

A preliminary scoping review exercise surveyed similar published studies through PROSPERO (International Prospective Register of Systematic Reviews), Ovid, and Google Scholar in January 2020. The first search was in June 2020 and an updated search was completed in August 2021. Search terms were manually generated and inputted onto the databases. Citation chaining was performed to allow for the forward and backward research trail of potentially relevant studies, where they were retrieved through the mentioned information sources. Results of the database search were catalogued in a referencing manager (EndNote X9) and then transferred to Rayyan where the records were de-duplicated. Finally, resulting records underwent the two stages of screening. To minimize language bias, included studies written in Chinese and Spanish in the first and second screenings were translated twice by different native-speaker researchers before deciding at the full-text screening stage.

### Data items

Study outcomes were within 1.5–24 months. We included all outcomes, coded and conceptually mapped them, and then categorized into four domains: (a) patient condition, (b) self-management, (c) social, and (d) delivery outcomes. Patient condition outcomes are related to the symptoms of the patient. Symptomatology, relapse rate, medication adherence, depression comorbidity, recovery and stabilization, rehospitalization, disability-adjusted life years, and cognitive functioning are categorized under this domain. Second, self-management outcomes denote the wellbeing and health promotion of the patient. It includes self-management outcomes that include self-care, knowledge about psychosis, quality of life, psychosocial functioning, and ability to seek medical consultation/help. Third, social outcomes are others-oriented and involve or affect their wider sphere, comprising of family environment, EE, social and occupational functioning, and psychosocial functioning. And fourth, delivery outcomes refer to intervention administration, included attendance rates and service user satisfaction.

### Study risk of bias assessment

We used the Integrated Quality Criteria for Review of Multiple Study Designs [ICROMS; [51]] to accommodate for the variability in design, and the Consolidated Health Economic Evaluation Reporting Standards [CHEERS; [52]] for the two economic evaluations. ICROMS work as a point system according to study-specific quality criteria based on a decision matrix, where mandatory items and a minimum score for each study design are added to reach a decision for the study. For the CHEERS checklist, the markings are 1 score for *Yes* if it is reported, 0 for *No* if otherwise, and 0.5 for partially reported. NA indicates not applicable. The scores are tallied and averaged after omitting counts for NA. The midpoint, described as *average*, is 17 of the 24 items. All studies were included regardless of scores.

### Data extraction and analysis

A data extraction table was developed based on the Cochrane Data Collection Form for Intervention Reviews: RCTs and non-RCTs and from the data headings of Sin et al. [53]. Information was extracted from each included study on: (A) study specifics (authors, publication year, study source, geographical context, study design and study aim); (B) participants (number of participants and attrition rate, age range, diagnosis); (C) theoretical basis (general underpinning theory or concept, specific theory or rationale behind approach); (D) intervention specifications (delivery platform, delivery agents, intervention program specifics, and recommendations); and (E) outcome measures (outcome definition, time points measured, outcome results).

A narrative approach was used to synthesize the data [54]. Synthesis was performed after extracting data from the included articles to remain inclusive in reviewing the records. Because of the heterogeneity across studies, further analysis was performed using the thematic framework as an interpretive method to categorize information from variable studies to maximize generation and exploration of overarching themes according to the research questions [55]. These stages of fractioning the main themes of the results through codes, and then clustering them into intervention features and strategies, aimed to highlight active ingredients from different the family-based interventions.

## Results

### Study characteristics

**Fig. 1:**
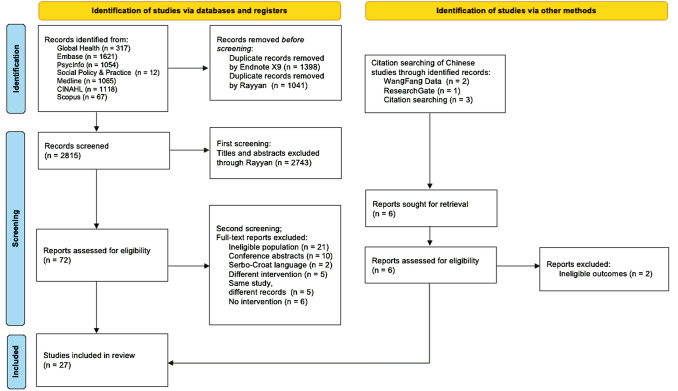
PRISMA Flow Diagram. From: Page MJ, McKenzie JE, Bossuyt PM, Boutron I, Hoffmann TC, Mulrow CD, et al. The PRISMA 2020 statement: an updated guideline for reporting systematic reviews. BMJ 2021;372:n71. https://doi.org/10.1136/bmj.n71. For more information, visit: http://www.prisma-statement.org/

Resulting studies from database searches were 5254 records, with 2815 titles and abstracts for the first screening phase and 72 full-text articles. There were four records related to one study (Study 1), including a doctoral dissertation, two articles written in Chinese and one article in English journals that reported the same study aims and outcomes, therefore the first English publication (Study 1) was selected for this review while the rest were excluded. Two other non-English articles were in Serbo-Croat but could not be translated. We were open to including conference abstracts to the review, but only contained limited information. Records that did not deliver the intervention were excluded as well. Lastly, we excluded a PhD dissertation and three more records because it reported the same information as the core study. This rationale applies to study 21 which had a 14-year follow-up record. Published journal articles and one book chapter were included in this review. This led to 27 studies (with corresponding numbers 1–27 listed in Table 1). Figure 1 illustrates the PRISMA flow chart diagram for the process and reasons for exclusion.

**Table 1:** Family-Based Intervention specifications

Study no.	Study	Geographical context	Study	Sample Size (PWP)	Conceptual/theoretical basis	Family intervention features	Delivery platform and agent	Outcome measures	Time Points (months**)**
1	Li and Arthur [56]	Beijing, China	RCT	101	Psychoeducation; EE	Psychoeducation for patient and family	Outpatient; nurses	Symptom severity, Psychosocial functioning, EE in family/family dynamics	9
2	Alibeigi and Momeni [57]	Tehran, Iran	RCT	67	Minkowitz Family-Focused Treatment Package; EE	Group family therapy held in 12 weekly sessions	Outpatient; clinical psychologists, psychiatrists	Symptomatology, psychosocial functioning	3
3	Barekatain et al. [58]	Isfahan, Iran	RCT	123	Aftercare family support through task-sharing; psychoeducation	Psychoeducation sessions (> 6) for family Weekly follow-up calls and monthly home visits for patient and family	Community, Inpatient; Chief psychiatrist, and 2 consultant psychiatrists	Symptom severity, re-hospitalization rate	12
4	Cai [59]	Shanghai, China	RCT	256	Family-Directed Cognitive Adaptation for Schizophrenia (Friedman-Yakoobian et al., 2009)	Comprehensive Family Therapy to patients and family Psychoeducation for family members	Community; Psychiatric health workers	Cognitive functioning, symptom severity	18
5	Khalil et al. [60]	Cairo, Egypt	RCT	60	Behavioral Family Psychoeducation Program (BFPEP)	Culturally Adapted BFPEP: engagement (1 session), assessment (1 session), psychoeducation for family (3 sessions), communication enhancement training (4 sessions), problem-solving skills training (4 sessions), termination (1 session)	Outpatient; Researchers trained for behavioral family therapy	Symptom severity, quality of life, social functioning, medication adherence	9
6	Xiong et al. [61]	Shashi and Jingzhou China	RCT	63	Talking therapy and family intervention theories	Family intervention done in three phases: Introductory phase (2–3 meetings); Treatment phase with monthly 45-min patient counselling sessions and monthly 90-min family sessions with psychoeducation and therapy components; Maintenance phase within family group meetings	Outpatient; Members of the PWP’s community	Symptom management, social functioning and integration, coping strategy, medication reduction	24
7	Xiang et al. [62]	Sichuan, China	RCT	80	Community care	Psychoeducation After-care network set-up (e.g., family seminars and workshops)	Communities; Village doctors, psychiatrists	Medication adherence; understanding of and changing attitude towards mental disease; effectiveness of clinical treatment; improvement of the patients' working ability; decrease in the rate of social disturbance	4
8	Rami et al. [63]	Cairo, Egypt	RCT	60	Behavioral Family Psychoeducational Program (BFPEP)	Behavioral Family Psychoeducational Program (BFPEP) with the following components: 1) 14 one-hour individual family therapy sessions over 6 months, and 2) Psychoeducation modules on psychoeducation for PWP and family (5 sessions), communication enhancement training (4 sessions), and problem-solving skills training (4–5 sessions)	Outpatient clinics of the Institute of Psychiatry Ain Shams University Hospitals; Family members and researchers	Rate of improvement of clinical variables including the patient’s social functions, medication adherence, and quality of life	6
9	Zhang et al. [64]	Jiangsu, China	RCT	83	Family as after-care	Psychoeducation for PWP and family for 3 months and individual family counselling as the need arises. Home visits were done for those who cannot attend	Outpatient; Psychiatric health workers, attending physicians, counsellors	Increased medication adherence	18
10	Ngoc et al. [65]	Da Nang, Vietnam	RCT	59	Family Schizophrenia Psychoeducation Program (FSPP; Kung et al., 2012)	Adapted FSPP Medication	Inpatient; Psychiatrist, 2 psychologists and 2 nurses	Quality of life, medication adherence	6
11	Qiu et al. [66]	Shandong, China	RCT	112	Psychological and behavioral education theories	Psychoeducation with family (4 lectures) Home visits to facilitate family communication, after-care training, consultations, on-call availability in case of emergencies, and mutual support network with other families	Outpatient and community; Trained psychiatrists	Quality of life	6
12	Husain et al. [67]	Karachi, Pakistan	RCT	36	Culturally adapted psychosocial family intervention in Pakistan (Naeem et al., 2015; Husain et al., 2017)	*1*0 sessions (40–60 min each) for the first 8 weeks and fortnightly for the remaining 4 weeks with the following program components: 1. Psychoeducation 2. Cognitive-behavioural skills training for stress-management, coping and problem-solving 3. Crisis intervention and suicide risk management 4. Relapse prevention 5. Education and support regarding the family environment, including communication training	Outpatient mental health services; Trained research clinician	Symptom severity, social and occupational functioning, depression comorbidity	*3*
13	Yang and Pearson [68]	Beijing, China	Qualitative	1	Eclectic structural family therapy; Psychoeducation (therapist's role)	Clinical individual and family psychotherapy	Outpatient; Clinical psychologist	Management of symptoms through EE, recognition of negative symptoms, minimized presenting problem	16
14	Asmal et al. [69]	Stellenbosch, South Africa	Qualitative	20	Multi-family Group Model	Psychoeducation for family based on a semi-structured 90 min. sessions fortnightly	University of Stellenbosch; Psychiatrist, nurse of > 20 years of experience, qualitative researchers	Level of EE, symptom severity	3
15	van der Geest [70]	Matagalpa, Nicaragua	Qualitative	Not specified	Family support; Face-to-face psychoeducation	Psychoeducation for patient and family Emotional support through home visits	NGO; Psychiatrist, nurses, community volunteers	Quality of life, emotional support	NA
16	Palmeira et al. [71]	Rio de janeiro, Brazil	Qualitative	24	Problem-solving therapy through family therapy; Recovery program by immediate community	“*Entrelaços*” Peer Support Program: integrates psychoeducation and problem-solving therapy through multi-family groups	Outpatient; Hospital staff, families	Knowledge of schizophrenia, self-care know-how	18
17	Devaramane et al., [72]	Mangalore, India	NCBA	18	Adapted Brief Family Intervention (Varghese et al., 2002)	Psychotherapy	In- and outpatient; MH professionals	Symptom severity, patient’s perceived level of EE	3
18	Thara et al. [73]	Chennai, India	NCBA	26	Family Education Program (Goldstein, 1995)	Structured Psychoeducation Program (6 sessions) with film-showing on family care, empowerment, and support; interactive follow-up sessions with professionals Informal Psychoeducation Program that met regularly to reinforce concepts previously learned	NGO; NGO director, consulting psychiatrist, case managers	Symptom severity	24
19	Padmavathi et al. [74]	Karnataka, India	NCBA	2	Family-focused therapy (Miklowitz and Chung, 2016); Family psychoeducation; Family systems approach	12 sessions of family-focused therapy and psychoeducation for patient and family carer; 5–8 sessions of communication enhancement training through video demonstration, observation of family dynamics and problem-solving skills training	Inpatient at the psychiatric unit of National Institute of Mental Health and Neurosciences (NIMHANS), Bengaluru; Therapist as facilitator, Test rater	Symptom severity that affect the social and occupational functioning	10
20	Sharma et al. [75]	Delhi, Noida, and Ghaziabad, India	NCBA	40	Psychoeducation Intervention Package	5 psychoeducation sessions (with 7–10 day intervals between sessions) for parents where they were also communication skills improvement. Family carers were taught to prioritize their mental health	Outpatient, multi-site; Researcher	Symptomatology	*Over 1.5*
21	Ran et al. [76]	Chengu, China	cRCT	357	Psychoeducational Family Approach (Anderson et al. 1986); Vulnerability- Stress Model (Lalonde 1995)	Psychoeducation—family education once a month for 9 months, quarterly multiple family workshops, and crisis intervention when necessary Medication	Communities; 15 independent researchers, local village broadcast network	Symptom severity, relapse rates	9
22	Zhang et al. [77]	Jinan and Shanghai, China	cRCT	1048	Psychoeducational Family Intervention	Group psychotherapy that included 14 psychoeducation lectures and five group discussions	Communities; Trained psychiatrists, nurses	Relapse rate, rate of regular work	12
23	Zhang et al., [78]	Jinan, Hangzhou, Shengyang, Suzhou, and Shanghai, China	cRCT	3092	Family psychoeducation; After-care task shared with family	Family Education Program with 8 lectures and 3 group discussions	Multi-site communities; Research team	Recovery rate, symptom severity (negative symptoms), relapse rate	12
24	Rahayu et al. [79]	Sulawesi, Indonesia	CBA	78	Individual cognitive therapy; Family therapy	Cognitive therapy (3 sessions) for patients Family therapy through psychoeducation sessions (6 sessions, 30–45 min each)	Orphanage; Psychiatric nurses	Decreased prodromal psychosis symptomatology; increased self-esteem	3
25	Zhao et al. [80]	Hunan, China	CBA	31	Assertive Community Treatment; McFarlane Family Psychoeducational Model	Family-based Assertive Community Treatment (ACT): 1) 2–3 home visits to deliver ACT care, 2) 2-h psychoeducation sessions fortnightly for 24 weeks, and 3) Mutual Support Group Program for PWP and family	Community; Psychiatrist, psychiatric nurses, clinical psychologist	Symptomatology; Social, personal, and everyday functioning	12
26	Anh et al. [81]	Vietnam	Economic Evaluation	Schizophrenia prevalence in Vietnam (2008)	NA	Health education and communication for patients and their families to create an environment without criticism and stigma	NA	DALYs averted	NA
27	Phanthunane et al. [82]	Thailand	Economic Evaluation	Patients with schizophrenia in Thailand	NA	10 weekly 2-h sessions, 2 booster sessions for patients and family every year over a patient’s lifetime	Psychiatric nurse	Health outcomes in DALYs	NA

*BFPEP* brief family psychoeducation program, *CBA* controlled before-after study, *cRCT* cluster randomized control study, *EE* expressed emotion, *DALYs* disability-adjusted life years, *FSPP* family schizophrenia psychoeducation program, *MH* mental health, *NCBA* non-controlled before–after study, *NGO* non-government organization, *PWP* person with psychosis, *QoL* quality of life, *RCT* randomized control trial study

### Overview of included studies

Study characteristics of individual studies are presented in Table 1. Study designs [52] were individual level randomized control trials [44%, studies 1–12; [56–67]], qualitative studies [15%, i.e., studies 13–14 [68, 69] are case studies of individual families and studies 15–16; [70, 71] are descriptive studies of specific programs], non-controlled studies [15%, studies 17–20; [72–75]], cluster randomized control trials [11%, studies 21–23; [76–78]], controlled studies [7%, studies 24–25; [79, 80]], and cost-effectiveness analyses [7%, studies 26–27; [81, 82]]. Two of the included articles are in Chinese (studies 1 and 11) and the rest are in English. From the 27 included studies, 11 were conducted in China (Studies 1, 4, 6–7, 9, 11, 13, 21–23, and 25); four studies from India, (studies 17–20); two each from Iran (studies 2–3), Vietnam (studies 10 and 26), and Egypt [5, 8]; and one study each from South Africa (study 14), Brazil (study 16), Indonesia (study 24), Thailand (study 27), Nicaragua (study 15), and Pakistan (study 12).

Geographical settings were distributed in urban, rural, and multisite settings. Sixty-nine percent of the intervention sites were in rural settings (e.g., studies 14, 19, 24) and four studies were multisite (e.g., studies 6 and 20). Seventy-four percent were based on community sites (i.e., for home visits; seven studies), as outpatient (e.g., studies 21–23), as combination of in- and out-patient facilities (studies 3 and 17), and conducted within non-government organizations where people with psychosis resided (studies 15 and 18), and an orphanage (study 24). Twenty of the 27 studies included participants diagnosed with schizophrenia, three studies included participants with schizophrenia and bipolar disorder (studies 2–3 and 16), one included participants with schizophrenia and schizoaffective disorders (study 7), and one included participants with bipolar disorder (study 19).

### Synthesis of results

Results were coded and narratively synthesized according to an emergent thematic framework based on the research objectives to describe family-based interventions and to explore how this is evidenced in LMICs. They were clustered into intervention features with the study outcomes, as well as the delivery strategies in different LMIC contexts.

#### Intervention features

The conceptual or theoretical underpinnings are the rationale for implementing family-based interventions. Two overarching themes emerged: psychotherapeutic components and task-sharing. Psychotherapeutic components address symptoms of psychosis psychologically, i.e., through processing or talking about mental and emotional distress. In the studies, three main components were salient: psychoeducation, therapeutic technique, and family systems approach. Twenty-four studies cited psychotherapeutic components as the basis for family-based intervention (studies 1–8, 10–25), 22 of which were based on psychoeducation (studies 1, 3–12, 14–16, 18–25). Twelve studies utilized therapeutic techniques, specifically, three studies employed individual therapy for family members with psychosis (studies 6, 13, and 24) and nine studies utilized family therapy (studies 2, 4, 6, 8–9, 13, 17, and 24–25), with one study that had multiple family therapy (study 22). Format of psychoeducation sessions included workshops (e.g., studies 5 & 12) and interactive discussions (e.g., study 6 & 18) within the time frames of three to 14 regular lectures lasting 15 min to two hours. The content generally includes a series of lectures about schizophrenia (e.g., study 11), different treatments and rehabilitation (e.g., study 21), caring for a family member with psychosis (e.g., 9), coping strategies, and how to care for the carers (e.g., study 22). Second, the family systems approach highlights the dynamics within the family, addressing the interaction among members to affect the outcomes of the person with psychosis. Studies specifically mentioned expressed emotion (EE; e.g., study 19) and communication/interaction patterns. For example, Zhang [77] aimed to decrease family stress and EE, thereby reducing relapse rates. Yang and Pearson [68] proposed to manage symptoms by reducing EE, and Asmal et al. [69] associated greater family support to reduced EE. Finally, task-sharing in this review pertains to assigning care and support by family members, to provide quality aftercare (e.g., study 3) and community networking, such as care networks (study 7) and social support networks (studies 2 and 18). Aftercare was a feature to strengthen the patient–family relationship and to provide support for other families in similar situations. Four studies (studies 3, 9, 11, and 15) employed home visits either to encourage sustained participation in the intervention (e.g., study 9) or to perform home-based therapeutic support (e.g., study 15).

Family-based interventions in LMICs reported multiple outcomes for each study. Among the 27 studies, patient condition was mentioned 46 times from 26 studies, all reporting positive health impacts. For example, study 23 reported three patient outcome measures: recovery rate, symptom severity (negative symptoms), and relapse rate. Only studies 3, 12, and 18 reported no change for their specified patient conditions. Second, self-management outcomes appeared 17 times from 11 studies, all reporting positive health impacts. Study 16 for instance measured increased knowledge about schizophrenia and self-care skills. Third, social outcomes were mentioned 12 times from 15 studies, with studies 3, 12 and 13 reporting no change in social outcomes. Lastly, three studies (studies 10, 14 and 26) presented positive delivery outcomes, measuring an increase in attendance rates and service user satisfaction.

#### Delivery strategies

All the studies had more than two delivery agents for the intervention, except for one clinical study with one clinical psychologist in the individual family sessions [68]. Sixty-eight percent of the interventions were employed by mental health professionals, specifically, psychiatrists, clinical psychologists or therapists, and psychiatric nurses, while the rest were employed by social workers, researchers, and non-government organization staff. Intervention endpoints ranged from three to 24 months. Seventy-four percent of the studies cited their own preliminary research on the topic (e.g., studies 17–18, 26) and population (e.g., study 8), and robustness of the method (e.g., study 22) as contributing factors to implementation. One-third of the studies underscored the importance of sustained family engagement in family-based interventions (e.g., studies 4 and 13), and a quarter attributed favorable delivery to cultural adaptation (e.g., studies 2 and 12). On the other hand, the studies also mentioned challenges in implementation, particularly those that lack robustness of research method (e.g., studies 8 & 10), including stigma held by family and community members (e.g., studies 7 and 27), and waning family involvement (studies 6 and 21). Lastly, authors of the studies offered improvements to the evaluations, such as longer follow-ups (e.g., study 3), and to the interventions, such as to use less and briefer sessions (e.g., study 26), to integrate in routine clinical settings (e.g., study 2), to task-share care and obtain support from family (e.g., study 24), to ensure cultural appropriateness (e.g., study 13), and to allocate a public health budget for it (e.g., studies 15, 26 and 27).

#### Quality assessment

Six studies (studies 12–15 and 26–27) had low risk of bias via ICROMS (Table 2) and CHEERS checklist (Table 3). Two studies yielded moderate risk of bias (studies 3–4), i.e., minimum ICROMS scores were met but mandatory scores were unmet. High risk of bias was indicated for 70% of the studies (studies 1–2, 5–11, and 16–25), i.e., minimum and mandatory scores in the ICROMS were unmet.

**Table 2:** Risk of bias assessment using ICROMS for majority of the studies

Study number	Study	Study design	Dimension							Total score	Minimum score* met	Mandatory criteria met
			(1) Clear aims and justification	(2) Managing bias in sampling and between groups	(3) Managing bias in outcome measurement and blinding	(4) Managing bias in follow-up	(5) Managing bias in other study aspects	(6) Analytical rigour	(7) Managing bias in reporting/ethical considerations			
1	Li and Arthur [56]	RCT	2	3	3	3	1	0	7	19	No	No
2	Alibeigi and Momeni [57]	RCT	2	2	4	2	2	1	7	20	No	No
3	Barekatain et al. [58]	RCT	2	2	6	4	1	2	6	23	Yes	No
4	Cai [59]	RCT	2	3	6	5	4	2	4	26	Yes	No
5	Khalil et al. [60]	RCT	2	4	3	0	1	1	6	17	No	No
6	Xiong et al. [61]	RCT	2	2	4	4	2	1	4	19	No	No
7	Xiang et al. [62]	RCT	2	1	5	3	1	1	0	13	No	No
8	Rami et al. [63]	RCT	2	1	3	6	2	1	6	21	No	No
9	Zhang et al. [64]	RCT	2	1	3	5	1	1	4	17	No	No
10	Ngoc et al. [65]	RCT	2	3	2	2	1	1	5	16	No	No
11	Qiu et al. [66]	RCT	2	2	3	4	2	1	3	17	No	No
12	Husain et al. [67]	RCT	2	4	4	5	2	1	9	27	Yes	Yes
13	Yang and Pearson [68]	Qualitative	6	2	2	2	2	2	6	22	Yes	Yes
14	Asmal et al. [69]	Qualitative	6	2	2	2	1	1	8	22	Yes	Yes
15	van der Geest [70]	Qualitative	4	2	1	1	1	1	7	17	Yes	Yes
16	Palmeira et al. [71]	Qualitative	6	2	1	1	0	1	1	12	No	No
17	Devaramane et al. [72]	NCBA	4	0	4	2	2	1	9	22	No	No
18	Thara et al. (2005)	NCBA	4	2	2	1	4	1	6	20	No	Yes
19	Padmavathi et al. [74]	NCBA	6	2	0	1	1	1	1	12	No	Yes
20	Sharma et al. [75]	NCBA	1	0	6	2	1	2	5	17	No	No
21	Ran et al. [76]	cRCT	2	2	4	4	1	1	5	19	No	No
22	Zhang et al., [77]	cRCT	2	3	3	6	1	1	5	21	No	No
23	Zhang et al., [78]	cRCT	2	2	3	5	2	1	2	17	No	No
24	Rahayu et al., [79]	CBA	2	0	5	1	1	1	3	13	No	No
25	Zhao et al., [80]	CBA	2	0	5	1	1	1	7	17	No	No

*CBA* controlled before–after study, *cRCT* cluster randomized control study, *NCBA* non-controlled before–after study, *RCT* randomized control trial study

**Table 3:** Risk of Bias Assessment Using CHEERS Checklist for Included Economic Evaluation Studies Assessment

	Anh et al. [81]	Phanthunane et al. [82]
Title and abstract		
Title	Y	Y
Abstract	Y	Y
Introduction		
Background and objectives	Y	Y
Methods		
Target population and subgroups	Y	Y
Setting and location	Y	Y
Study perspective	Y	Y
Comparators	Y	Y
Time horizon	Y	Y
Discount rate	Y	Y
Choice of health outcomes	Y	Y
Measurement of effectiveness	Y	Y
Measurement and valuation of preference-based outcomes	Y	Y
Estimating resources and costs	Y	Y
Currency, price date, and conversion	Y	Y
Choice of model	Y	Y
Assumptions	Y	Y
Analytical methods	Y	Y
Results		
Study parameters	Y	Y
Incremental costs and outcomes	Y	Y
Characterizing uncertainty (single-study economic evaluation)	NA	NA
Characterizing uncertainty (model-based economic evaluation)	Y	Y
Characterizing heterogeneity	NA	NA
Discussion		
Study findings, limitations, generalizability, and current knowledge	Y	Y
Other		
Source of funding	NA	Y
Score	21/21	22/22
Reporting quality based on % score	Good	Good

## Discussion

To the best of our knowledge, this is the first review that synthesized the various family-based interventions in LMICs. It aimed to describe intervention features with their study outcomes, to identify the delivery strategies within the different LMIC contexts, and to appraise this evidence.

### Summary of findings

Almost all studies examined family-based intervention as a stand-alone complex intervention; with only two studies utilizing antipsychotic meditation alongside family-based intervention (studies 10 and 21). Between 1993 and 2021, it appears that there was an increased delivery in LMICs, with 60% of the recorded evidence from the recent decade (2011–2020). It is encouraging to see an increase in number of reported studies, which also varied in design: trials and noncontrolled intervention studies, qualitative research, and economic evaluation from national data. While it appears ripe to adopt this intervention to low-resource contexts, systematic and scientific approach to planning it is essential. Most of the trials and before–after studies had methodological issues in randomization and in minimizing reporting bias. Despite this constraint, family-based intervention was still recommended by all included studies because of its cited effectiveness in individual studies, primarily in symptom reduction and improved family dynamics. A majority of the studies also reported decreased relapse rate, consistent with the evidence from HICs [33, 53, 83]. Additionally, patient outcomes related to decreased EE [20, 22, 84] that have been observed in HICs were also evident in the LMICs. It seems universal that being mindful of EE within the family unit could optimize involvement of family members when aftercare is dependent on them, increasing the quality of task-sharing from family members.

Even with the diversity of the geographical settings, key features in the family-based interventions were also similar to those administered in HICs, particularly psychoeducation and therapeutic components. Psychoeducation was the most common intervention feature in delivering family-based intervention across the different kinds of studies and contexts, which could be implemented in different locations and by at least one delivery agent (i.e., by a psychologist in a psychotherapy session). This may not be surprising for psychosis interventions in HICs, but it is notable that this review highlights psychoeducation for utilizing family-based intervention in LMICs, coinciding with mhGAP recommendations for priority interventions for psychosis [85–87]. Moreover, recent suggestions to fill in gaps for early psychosis intervention and research involve the family [7, 88]. Finally, cultural appropriateness was identified as an essential foundation in intervention features and implementation, consistent with previous literature on implementing family- and community-based interventions in LMICs [9, 89–92]. This review illustrated how anecdotes and stories in psychoeducation workshops were adapted for religious and cultural considerations as well as Western theoretical and conceptual bases contextualized to suit their population and therapeutic aims.

### Strengths and Limitations

Our inclusion criteria captured and represented all relevant studies from LMICs. In addition, language bias was minimized by screening Spanish and Chinese articles and eventually including a Chinese-written article, but we were unable to translate two publications in Serbo-Croat found in the first screening. Synthesis was obtained narratively because of the variability of study outcomes. Three RCTs with low risk bias may not be enough to meaningfully pool them and would not satisfy the objectives of representing the current state of family-based interventions in LMICs. Further analysis could investigate the direction of effectiveness through the effect direction plot, suggested by Cochrane [93, 94]. One limitation that developed in the analysis phase was that the included studies are classified as lower- or upper-middle-income countries, thus indicative of a research gap in the records of family-based intervention for psychosis in low-income countries. Lastly, majority of the included studies had a moderate to high risk of bias mainly due to their methodological quality, therefore conclusions about the effectiveness of this intervention is limited by this. Recommendations from the studies for scaling up include quality research methodology and further research work.

### Directions for practice and research

The evidence from this review suggests that participation of the family caregiver was essential in facilitating the family-based intervention in LMICs. Since up to 90% of aftercare is from family [5, 12, 95], whether by choice or convenience, the quality of engagement of the family caregiver with the person with psychosis is significant. The impetus to adapt family-based intervention in community-based rehabilitation in LMICs has been suggested and there seems to be a momentum in adopting and endorsing it in various parts of the world [8, 96]. Therefore, an inventory of the evidence of family-based interventions in LMICs contributes both in research and in practice. Contributing factors in delivery, such as brief and culturally adapted intervention features and advanced and formative methodological preparations, mentioned in this review can be considered for implementing family-based programs. Task-sharing within the family for example, has augmented out-patient care and medication adherence [88]. LMIC-based mental health interventions and practice can tap into the family as an underutilized resource [97] and build their capacities as allies in providing quality care, while they also are taught techniques to care for themselves.

Thus far, findings suggest that family-based intervention was implemented in communities and mostly outpatient facilities and can be delivered by at least one mental health professional or non-professionals. Consistent to general recommendations in family-based approaches in mental health intervention [10, 21], recommendations to engage policymakers (studies 8, 13, 15, 27) and to advance investment on this intervention (studies 15, 26–27) potentially enable scale-up. Active ingredients of delivered family-based interventions in LMICs fundamentally share key elements with the general literature, particularly on the content of sessions (e.g., psychoeducation, skills training), adapting to the cultural settings of the family, and a less rigid delivery strategy (e.g., time frame can be from months to years and delivery agent, and cooperation of a family member), and therapeutic techniques (e.g., talking therapies and family systems approach) [98]. While presenting the current state of family-based interventions in LMICs may prove significant, it would be worthwhile to add contextual definitions of effectiveness to the discourse.

## Conclusion

A salient theme that emerged was the methodological strengths and weaknesses of the included studies and how these appear to impact the delivery of family-based interventions. Recommendations to refine interventions that involve the family can be gathered from this review. The evidence presented can further provide more definitive information on how to overcome barriers to implementation in LMICs. Large randomized controlled trials could provide more decisive evidence but depending on the context, careful consideration should be evaluated for the feasibility of conducting these. Cultural adaptation contributes to implementing this in LMIC contexts, and perhaps a challenge could also be in the conceptualization phase of the intervention, utilizing indigenous and evolving concepts of family structure and support, as well as contextualizing aftercare and recovery, to strengthen features of family-based interventions. Adopting a culturally grounded family-based intervention strategy can be a foundation to facilitate a robust community-based rehabilitation. Taken together, these findings may inform policymakers, healthcare providers, and academics to improve patient outcomes through a cost-effective intervention that can promote more effective task-sharing of quality care with the family.

## Supplementary Information

Below is the link to the electronic supplementary material.

Supplementary file1 (DOCX 40 kb)
