# Supplementary material for: Exploring the effectiveness of family-based interventions for psychosis in low- and middle-income countries: a systematic review
Source: Soc Psychiatry Psychiatr Epidemiol. 2022 Jun 14;57(9):1749–69. doi: 10.1007/s00127-022-02309-8 (PMC9375736; doi:10.1007/s00127-022-02309-8)
Supplement: Supplementary file 1 — Supplementary file1 (DOCX 40 kb) [file 127_2022_2309_MOESM1_ESM.docx]

**Appendix A. PRISMA Checklist**

| **Section and Topic** | **Item #** | **Checklist item** | **Location where item is reported*** |
| --- | --- | --- | --- |
| **TITLE** | | |  |
| Title | 1 | Identify the report as a systematic review. | 1 |
| **ABSTRACT** | | | 1 |
| Abstract | 2 | See the PRISMA 2020 for Abstracts checklist. |  |
| **INTRODUCTION** | | |  |
| Rationale | 3 | Describe the rationale for the review in the context of existing knowledge. | 2 |
| Objectives | 4 | Provide an explicit statement of the objective(s) or question(s) the review addresses. | 2 |
| **METHODS** | | |  |
| Eligibility criteria | 5 | Specify the inclusion and exclusion criteria for the review and how studies were grouped for the syntheses. | 2 |
| Information sources | 6 | Specify all databases, registers, websites, organisations, reference lists and other sources searched or consulted to identify studies. Specify the date when each source was last searched or consulted. | 2, 3 |
| Search strategy | 7 | Present the full search strategies for all databases, registers and websites, including any filters and limits used. | 2,  Appendix B |
| Selection process | 8 | Specify the methods used to decide whether a study met the inclusion criteria of the review, including how many reviewers screened each record and each report retrieved, whether they worked independently, and if applicable, details of automation tools used in the process. | 2 |
| Data collection process | 9 | Specify the methods used to collect data from reports, including how many reviewers collected data from each report, whether they worked independently, any processes for obtaining or confirming data from study investigators, and if applicable, details of automation tools used in the process. | 2,3 |
| Data items | 10a | List and define all outcomes for which data were sought. Specify whether all results that were compatible with each outcome domain in each study were sought (e.g. for all measures, time points, analyses), and if not, the methods used to decide which results to collect. | 3 |
|  | 10b | List and define all other variables for which data were sought (e.g. participant and intervention characteristics, funding sources). Describe any assumptions made about any missing or unclear information. | 3 |
| Study risk of bias assessment | 11 | Specify the methods used to assess risk of bias in the included studies, including details of the tool(s) used, how many reviewers assessed each study and whether they worked independently, and if applicable, details of automation tools used in the process. | 3 |
| Effect measures | 12 | Specify for each outcome the effect measure(s) (e.g. risk ratio, mean difference) used in the synthesis or presentation of results. | 3 |
| Synthesis methods | 13a | Describe the processes used to decide which studies were eligible for each synthesis (e.g. tabulating the study intervention characteristics and comparing against the planned groups for each synthesis (item #5)). | 3 |
|  | 13b | Describe any methods required to prepare the data for presentation or synthesis, such as handling of missing summary statistics, or data conversions. | 3 |
|  | 13c | Describe any methods used to tabulate or visually display results of individual studies and syntheses. | 3 |
|  | 13d | Describe any methods used to synthesize results and provide a rationale for the choice(s). If meta-analysis was performed, describe the model(s), method(s) to identify the presence and extent of statistical heterogeneity, and software package(s) used. | 3 |
|  | 13e | Describe any methods used to explore possible causes of heterogeneity among study results (e.g. subgroup analysis, meta-regression). | NA |
|  | 13f | Describe any sensitivity analyses conducted to assess robustness of the synthesized results. | NA |
| Reporting bias assessment | 14 | Describe any methods used to assess risk of bias due to missing results in a synthesis (arising from reporting biases). | 3 |
| Certainty assessment | 15 | Describe any methods used to assess certainty (or confidence) in the body of evidence for an outcome. | 3 |
| **RESULTS** | | |  |
| Study selection | 16a | Describe the results of the search and selection process, from the number of records identified in the search to the number of studies included in the review, ideally using a flow diagram. | 3, Figure 1 |
|  | 16b | Cite studies that might appear to meet the inclusion criteria, but which were excluded, and explain why they were excluded. | 3 |
| Study characteristics | 17 | Cite each included study and present its characteristics. | 6 |
| Risk of bias in studies | 18 | Present assessments of risk of bias for each included study. | 7 |
| Results of individual studies | 19 | For all outcomes, present, for each study: (a) summary statistics for each group (where appropriate) and (b) an effect estimate and its precision (e.g. confidence/credible interval), ideally using structured tables or plots. | 7, Table 1 |
| Results of syntheses | 20a | For each synthesis, briefly summarise the characteristics and risk of bias among contributing studies. | 7, Tables 2 & 3 |
|  | 20b | Present results of all statistical syntheses conducted. If meta-analysis was done, present for each the summary estimate and its precision (e.g. confidence/credible interval) and measures of statistical heterogeneity. If comparing groups, describe the direction of the effect. | NA |
|  | 20c | Present results of all investigations of possible causes of heterogeneity among study results. | NA |
|  | 20d | Present results of all sensitivity analyses conducted to assess the robustness of the synthesized results. | NA |
| Reporting biases | 21 | Present assessments of risk of bias due to missing results (arising from reporting biases) for each synthesis assessed. | 7 |
| Certainty of evidence | 22 | Present assessments of certainty (or confidence) in the body of evidence for each outcome assessed. | NA |
| **DISCUSSION** | | |  |
| Discussion | 23a | Provide a general interpretation of the results in the context of other evidence. | 9 |
|  | 23b | Discuss any limitations of the evidence included in the review. | 9 |
|  | 23c | Discuss any limitations of the review processes used. | 9 |
|  | 23d | Discuss implications of the results for practice, policy, and future research. | 9, 10 |
| **OTHER INFORMATION** | | |  |
| Registration and protocol | 24a | Provide registration information for the review, including register name and registration number, or state that the review was not registered. | 2 |
|  | 24b | Indicate where the review protocol can be accessed, or state that a protocol was not prepared. | 2 |
|  | 24c | Describe and explain any amendments to information provided at registration or in the protocol. | 2 |
| Support | 25 | Describe sources of financial or non-financial support for the review, and the role of the funders or sponsors in the review. | 1 |
| Competing interests | 26 | Declare any competing interests of review authors. | 10 |
| Availability of data, code and other materials | 27 | Report which of the following are publicly available and where they can be found: template data collection forms; data extracted from included studies; data used for all analyses; analytic code; any other materials used in the review. | 10 |

*From:*  Page MJ, McKenzie JE, Bossuyt PM, Boutron I, Hoffmann TC, Mulrow CD, et al. The PRISMA 2020 statement: an updated guideline for reporting systematic reviews. BMJ 2021;372:n71. doi: 10.1136/bmj.n71. For more information, visit: <http://www.prisma-statement.org/>.

*NB: Page numbers in publication may slightly vary with manuscript.

**Appendix B. Search Terms for all databases**

Embase, MEDLINE, Global Health, PsycInfo, Social Policy and Practice, through the OVID platform

(Psychos?s or Brief Reactive Psychos?s or Bipolar disorder* or Schizoaffective Disorder* or Schizophreniform Disorder* or Schizophren* or Psychotic Disorder* or Severe mental illness* or Severe mental disorder* or Recent-onset psychos?s or Early onset psychos?s).mp. [mp=ti, ab, hw, tn, ot, dm, mf, dv, kw, fx, dq, nm, kf, ox, px, rx, an, ui, sy, bt, id, cc, tc, tm, sh, mh, pt]

(Family-based intervention* or Family therap* or Family-based or Parent* or Mother* or Father* or Primary care-giver* or Caregiver* or Guardian* or Sibling* or Brother* or Sister* or Home intervention* or Home-based intervention* or Family systemic therap*).mp. [mp=ti, ab, hw, tn, ot, dm, mf, dv, kw, fx, dq, nm, kf, ox, px, rx, an, ui, sy, bt, id, cc, tc, tm, sh, mh, pt] (2745719)

(afghanistan or albania or algeria or american samoa or angola or argentina or armenia or armenian or aruba or azerbaijan or bangladesh or republic of belarus or belarus or byelarus or belorussia or byelorussian or belize or british honduras or benin or dahomey or bhutan or bolivia or "bosnia and herzegovina" or bosnia or herzegovina or botswana or bechuanaland or brazil or brasil or bulgaria or burkina faso or burkina fasso or upper volta or burundi or urundi or cabo verde or cape verde or cambodia or kampuchea or khmer republic or cameroon or cameron or cameroun or central african republic or ubangi shari or chad or china or colombia or comoros or comoro islands or iles comores or mayotte or democratic republic of the congo or democratic republic congo or congo or zaire or costa rica or "cote d’ivoire" or "cote d’ ivoire" or cote divoire or cote d ivoire or ivory coast or cuba or djibouti or french somaliland or dominica or dominican republic or ecuador or egypt or united arab republic or el salvador or equatorial guinea or spanish guinea or eritrea or eswatini or swaziland or ethiopia or fiji or gabon or gabonese republic or gambia or "georgia (republic)" or georgian or ghana or gold coast or grenada or guam or guatemala or guinea or guinea bissau or guyana or british guiana or haiti or hispaniola or honduras or india or indonesia or timor or iran or iraq or jamaica or jordan or kazakhstan or kazakh or kenya or "democratic people’s republic of korea" or republic of korea or north korea or kosovo or kyrgyzstan or kirghizia or kirgizstan or kyrgyz republic or kirghiz or laos or lao pdr or "lao people's democratic republic" or lebanon or lebanese republic or lesotho or basutoland or liberia or libya or libyan arab jamahiriya or "macedonia (republic)" or macedonia or madagascar or malagasy republic or malawi or nyasaland or malaysia or malay federation or malaya federation or maldives or indian ocean islands or indian ocean or mali or micronesia or federated states of micronesia or kiribati or marshall islands or northern mariana islands or palau or tuvalu or mauritania or mexico or moldova or moldovian or mongolia or montenegro or morocco or ifni or mozambique or portuguese east africa or myanmar or burma or namibia or nepal or netherlands antilles or nicaragua or niger or nigeria or oman or muscat or pakistan or papua new guinea or new guinea or paraguay or peru or philippines or philipines or phillipines or phillippines or russia or russian federation or ussr or soviet union or union of soviet socialist republics or rwanda or ruanda or samoa or pacific islands or polynesia or samoan islands or navigator island or navigator islands or "sao tome and principe" or senegal or serbia or sierra leone or melanesia or solomon island or solomon islands or norfolk island or norfolk islands or somalia or south africa or south sudan or sri lanka or ceylon or saint lucia or "st. lucia" or "saint vincent and the grenadines" or saint vincent or "st. vincent" or grenadines or sudan or suriname or surinam or dutch guiana or netherlands guiana or syria or syrian arab republic or tajikistan or tadjikistan or tadzhikistan or tadzhik or tanzania or tanganyika or thailand or siam or timor leste or east timor or togo or togolese republic or tonga or tunisia or turkey or "turkey (republic)" or turkmenistan or turkmen or uganda or ukraine or uzbekistan or uzbek or vanuatu or new hebrides or venezuela or vietnam or viet nam or middle east or west bank or gaza or palestine or yemen or yugoslavia or zambia or zimbabwe or northern rhodesia or global south or africa south of the sahara or sub-saharan africa or subsaharan africa or africa, central or central africa or africa, northern or north africa or northern africa or magreb or maghrib or sahara or africa, southern or southern africa or africa, eastern or east africa or eastern africa or africa, western or west africa or western africa or west indies or indian ocean islands or caribbean or central america or latin america or "south and central america" or south america or asia, central or central asia or asia, northern or north asia or northern asia or asia, southeastern or southeastern asia or south eastern asia or southeast asia or south east asia or asia, western or western asia or europe, eastern or east europe or eastern europe or developing country or developing countries or developing nation? or developing population? or developing world or less developed countr* or less developed nation? or less developed population? or less developed world or lesser developed countr* or lesser developed nation? or lesser developed population? or lesser developed world or under developed countr* or under developed nation? or under developed population? or under developed world or underdeveloped countr* or underdeveloped nation? or underdeveloped population? or underdeveloped world or middle income countr* or middle income nation? or middle income population? or low income countr* or low income nation? or low income population? or lower income countr* or lower income nation? or lower income population? or underserved countr* or underserved nation? or underserved population? or underserved world or under served countr* or under served nation? or under served population? or under served world or deprived countr* or deprived nation? or deprived population? or deprived world or poor countr* or poor nation? or poor population? or poor world or poorer countr* or poorer nation? or poorer population? or poorer world or developing econom* or less developed econom* or lesser developed econom* or under developed econom* or underdeveloped econom* or middle income econom* or low income econom* or lower income econom* or low gdp or low gnp or low gross domestic or low gross national or lower gdp or lower gnp or lower gross domestic or lower gross national or lmic or lmics or third world or lami countr* or transitional countr* or emerging economies or emerging nation?).mp. [mp=ti, ab, hw, tn, ot, dm, mf, dv, kw, fx, dq, nm, kf, ox, px, rx, an, ui, sy, bt, id, cc, tc, tm, sh, mh, pt]

Cumulative Index to Nursing and Allied Health Literature (CINAHL)

"TX ( Psychosis or psychoses or Brief Reactive Psychosis or Bipolar disorder or Schizoaffective Disorder or Schizophreniform Disorders or Schizophrenia or schizophrenic or Psychotic Disorder* or Severe mental illness or Severe mental illnesses or Severe mental disorder* or Recent-onset psychosis or Recent-onset psychoses or Early onset psychosis or Early onset psychoses Psychosis or psychoses or Brief Reactive Psychosis or Bipolar disorder or Schizoaffective Disorder or Schizophreniform Disorders or Schizophrenia or schizophrenic or Psychotic Disorder* or Severe mental illness or Severe mental illnesses or Severe mental disorder* or Recent-onset psychosis or Recent-onset psychoses or Early onset psychosis or Early onset psychoses )

TX ( Family-based intervention* or Family therap* or Family-based or Parent* or Mother* or Father* or Primary care-giver* or Guardian* or Sibling* or Brother* or Sister* or Home intervention* or Home-based intervention* or Family systemic therap* )

TX ( afghanistan or albania or algeria or american samoa or angola or argentina or armenia or armenian or aruba or azerbaijan or bangladesh or republic of belarus or belarus or byelarus or belorussia or byelorussian or belize or british honduras or benin or dahomey or bhutan or bolivia or "bosnia and herzegovina" or bosnia or herzegovina or botswana or bechuanaland or brazil or brasil or bulgaria or burkina faso or burkina fasso or upper volta or burundi or urundi or cabo verde or cape verde or cambodia or kampuchea or khmer republic or cameroon or cameron or cameroun or central african republic or ubangi shari or chad or china or colombia or comoros or comoro islands or iles comores or mayotte or democratic republic of the congo or democratic republic congo or congo or zaire or costa rica or "cote d’ivoire" or "cote d’ ivoire" or cote divoire or cote d ivoire or ivory coast or cuba or djibouti or french somaliland or dominica or dominican republic or ecuador or egypt or united arab republic or el salvador or equatorial guinea or spanish guinea or eritrea or eswatini or swaziland or ethiopia or fiji or gabon or gabonese republic or gambia or "georgia (republic)" or georgian or ghana or gold coast or grenada or guam or guatemala or guinea or guinea bissau or guyana or british guiana or haiti or hispaniola or honduras or india or indonesia or timor or iran or iraq or jamaica or jordan or kazakhstan or kazakh or kenya or "democratic people’s republic of korea" or republic of korea or north korea or kosovo or kyrgyzstan or kirghizia or kirgizstan or kyrgyz republic or kirghiz or laos or lao pdr or "lao people's democratic republic" or lebanon or lebanese republic or lesotho or basutoland or liberia or libya or libyan arab jamahiriya or "macedonia (republic)" or macedonia or madagascar or malagasy republic or malawi or nyasaland or malaysia or malay federation or malaya federation or maldives or indian ocean islands or indian ocean or mali or micronesia or federated states of micronesia or kiribati or marshall islands or northern mariana islands or palau or tuvalu or mauritania or mexico or moldova or moldovian or mongolia or montenegro or morocco or ifni or mozambique or portuguese east africa or myanmar or burma or namibia or nepal or netherlands antilles or nicaragua or niger or nigeria or oman or muscat or pakistan or papua new guinea or new guinea or paraguay or peru or philippines or philipines or phillipines or phillippines or russia or russian federation or ussr or soviet union or union of soviet socialist republics or rwanda or ruanda or samoa or pacific islands or polynesia or samoan islands or navigator island or navigator islands or "sao tome and principe" or senegal or serbia or sierra leone or melanesia or solomon island or solomon islands or norfolk island or norfolk islands or somalia or south africa or south sudan or sri lanka or ceylon or saint lucia or "st. lucia" or "saint vincent and the grenadines" or saint vincent or "st. vincent" or grenadines or sudan or suriname or surinam or dutch guiana or netherlands guiana or syria or syrian arab republic or tajikistan or tadjikistan or tadzhikistan or tadzhik or tanzania or tanganyika or thailand or siam or timor leste or east timor or togo or togolese republic or tonga or tunisia or turkey or "turkey (republic)" or turkmenistan or turkmen or uganda or ukraine or uzbekistan or uzbek or vanuatu or new hebrides or venezuela or vietnam or viet nam or middle east or west bank or gaza or palestine or yemen or yugoslavia or zambia or zimbabwe or northern rhodesia or global south or africa south of the sahara or sub-saharan africa or subsaharan africa or africa, central or central africa or africa, northern or north africa or northern africa or magreb or maghrib or sahara or africa, southern or southern africa or africa, eastern or east africa or eastern africa or africa, western or west africa or western africa or west indies or indian ocean islands or caribbean or central america or latin america or "south and central america" or south america or asia, central or central asia or asia, northern or north asia or northern asia or asia, southeastern or southeastern asia or south eastern asia or southeast asia or south east asia or asia, western or western asia or europe, eastern or east europe or eastern europe or developing country or developing countries or developing nation? or developing population? or developing world or less developed countr* or less developed nation? or less developed population? or less developed world or lesser developed countr* or lesser developed nation? or lesser developed population? or lesser developed world or under developed countr* or under developed nation? or under developed population? or under developed world or underdeveloped countr* or underdeveloped nation? or underdeveloped population? or underdeveloped world or middle income countr* or middle income nation? or middle income population? or low income countr* or low income nation? or low income population? or lower income countr* or lower income nation? or lower income population? or underserved countr* or underserved nation? or underserved population? or underserved world or under served countr* or under served nation? or under served population? or under served world or deprived countr* or deprived nation? or deprived population? or deprived world or poor countr* or poor nation? or poor population? or poor world or poorer countr* or poorer nation? or poorer population? or poorer world or developing econom* or less developed econom* or lesser developed econom* or under developed econom* or underdeveloped econom* or middle income econom* or low income econom* or lower income econom* or low gdp or low gnp or low gross domestic or low gross national or lower gdp or lower gnp or lower gross domestic or lower gross national or lmic or lmics or third world or lami countr* or transitional countr* or emerging economies or emerging nation? ) AND TX ( Family-based intervention* or Family therap* or Family-based or Parent* or Mother* or Father* or Primary care-giver* or Caregiver* or Guardian* or Sibling* or Brother* or Sister* or Home intervention* or Home-based intervention* or Family systemic therap* ) AND TX ( Psychos?s or Brief Reactive Psychos?s or Bipolar disorder* or Schizoaffective Disorder* or Schizophreniform Disorder* or Schizophren* or Psychotic Disorder* or Severe mental illness* or Severe mental disorder* or Recent-onset psychos?s or Early onset psychos?s )

SCOPUS

psychosis OR psychoses OR ( "brief reactive psychosis" ) OR ( "bipolar disorder" ) OR ( "schizoaffective disorder" ) OR ( "schizophreniform disorders" ) OR schizophrenia OR schizophrenic OR ( "psychotic disorder*" ) OR ( "severe mental illness" ) OR ( "severe mental illnesses" ) OR ( "severe mental disorder*" ) OR ( "recent-onset psychosis" ) OR ( "recent-onset psychoses" ) OR ( "early onset psychosis" ) OR ( "early onset psychoses" ) AND ( "family-based intervention*" ) OR ( "family therap*" ) OR ( "family therap*" ) OR ( "family-based) or parent or mother or father or (" primary AND care-giver ") or guardian or sibling or brother or sister or (" home AND intervention* ") or (family systemic therap*" ) OR ( "home-based intervention*" )  AND afghanistan or albania or algeria or ("american samoa") or angola or argentina or armenia or armenian or aruba or azerbaijan or bangladesh or ("republic of belarus") or belarus or byelarus or belorussia or byelorussian or belize or ("british honduras") or benin or dahomey or bhutan or bolivia or ("bosnia and herzegovina") or bosnia or herzegovina or botswana or bechuanaland or brazil or brasil or bulgaria or ("burkina faso") or ("burkina fasso") or ("upper volta") or burundi or urundi or ("cabo verde") or ("cape verde") or cambodia or kampuchea or ("khmer republic") or cameroon or cameron or cameroun or ("central african republic") or ("ubangi shari") or chad or china or colombia or comoros or ("comoro islands") or ("iles comores") or mayotte or ("democratic republic of the congo") or ("democratic republic congo") or congo or zaire or ("costa rica") or ("cote d’ivoire") or ("cote d’ ivoire") or ("cote divoire") or ("cote d ivoire") or ("ivory coast") or cuba or djibouti or ("french somaliland") or dominica or ("dominican republic") or ecuador or egypt or ("united arab republic") or ("el salvador") or ("equatorial guinea") or ("spanish guinea") or eritrea or eswatini or swaziland or ethiopia or fiji or gabon or ("gabonese republic") or gambia or ("georgia republic") or georgian or ghana or ("gold coast") or grenada or guam or guatemala or guinea or ("guinea bissau") or guyana or ("british guiana") or haiti or hispaniola or honduras or india or timor or iran or iraq or jamaica or jordan or kazakhstan or kazakh or kenya or ("democratic people’s republic of korea") or ("republic of korea") or ("north korea") or kosovo or kyrgyzstan or kirghizia or kirgizstan or kyrgyz republic or kirghiz or laos or ("lao pdr") or ("lao people's democratic republic") or lebanon or ("lebanese republic") or lesotho or basutoland or liberia or libya or ("libyan arab jamahiriya") or ("macedonia republic") or macedonia or madagascar or ("malagasy republic") or malawi or nyasaland or malaysia or malay federation or ("malaya federation") or maldives or ("indian ocean islands") or ("indian ocean") or mali or micronesia or ("federated states of micronesia") or kiribati or ("marshall islands") or ("northern mariana islands") or palau or tuvalu or mauritania or mexico or moldova or moldovian or mongolia or montenegro or morocco or ifni or mozambique or portuguese east africa or myanmar or burma or namibia or nepal or (“netherlands Antilles”) or nicaragua or niger or nigeria or oman or muscat or pakistan or (“papua new guinea”) or (“new guinea”) or paraguay or peru or philippines or philipines or phillipines or phillippines or russia or (“russian federation”) or ussr or (“soviet union”) or (“union of soviet socialist republics”) or rwanda or ruanda or samoa or (“pacific islands”) or polynesia or (“samoan islands”) or (“navigator island”) or (“navigator islands”) or ("sao tome and principe") or senegal or serbia or (“sierra leone”) or melanesia or (“solomon island”) or (“solomon islands”) or (“norfolk island”) or (“norfolk islands”) or somalia or (“south Africa”) or (“south sudan”) or (“sri lanka”) or ceylon or (“saint lucia”) or ("st. lucia") or ("saint vincent and the grenadines") or (“saint Vincent”) or ("st. vincent") or grenadines or sudan or suriname or surinam or (“dutch Guiana”) or (“netherlands Guiana”) or syria or (“syrian arab republic”) or tajikistan or tadjikistan or tadzhikistan or tadzhik or tanzania or tanganyika or thailand or siam or (“timor leste”) or (“east timor”) or togo or (“togolese republic”) or tonga or tunisia or turkey or ("turkey republic") or turkmenistan or turkmen or uganda or ukraine or uzbekistan or uzbek or vanuatu or new hebrides or venezuela or vietnam or (“viet nam”) or (“middle east”) or (“west bank or gaza”) or palestine or yemen or yugoslavia or zambia or zimbabwe or (“northern rhodesia”) or (“global south”) or (“africa south of the sahara”) or (“sub-saharan africa”) or (“subsaharan africa”) or (“africa, central or central africa”) or (“africa, northern”) or (“north africa”) or (“northern africa”) or magreb or maghrib or sahara or (“africa, southern”) or (“southern africa”) or (“africa, eastern”) or (“east africa”) or (“eastern africa”) or (“africa, western”) or (“west Africa”) or (“western Africa”) or (“west indies”) or (“indian ocean islands”) or caribbean or (“central america”) or (“latin America”) or ("south and central america") or (“south America”) or (“asia, central”) or (“central asia”) or (“asia, northern”) or (“north asia”) or (“northern asia”) or (“asia, southeastern”) or (“southeastern asia”) or (“south eastern asia”) or (“southeast asia”) or (“south east asia”) or (“asia, western”) or (“western asia”) or (“europe, eastern”) or (“east Europe”) or (“eastern Europe”) or (“developing country”) or (“developing countries”) or (“developing nation?”) or (“developing population?”) or (“developing world”) or (“less developed countr*”) or (“less developed nation?”) or (“less developed population?”) or (“less developed world”) or (“lesser developed countr*”) or (“lesser developed nation?”) or (“lesser developed population?”) or (“lesser developed world”) or (“under developed countr*”) or (“under developed nation?”) or (“under developed population?”) or (“under developed world”) or (“underdeveloped countr*”) or (“underdeveloped nation?”) or (“underdeveloped population?”) or (“underdeveloped world”) or (“middle income countr*”) or (“middle income nation?”) or (“middle income population?”) or (“low income countr*”) or (“low income nation?”) or (“low income population?”) or (“lower income countr*”) or (“lower income nation?”) or (“lower income population?”) or (“underserved countr*”) or (“underserved nation?”) or (“underserved population?”) or (“underserved world”) or (“under served countr*”) or (“under served nation?”) or (“under served population?”) or (“under served world”) or (“deprived countr*”) or (“deprived nation?”) or (“deprived population?”) or (“deprived world”) or (“poor countr*”) or (“poor nation?”) or (“poor population?”) or (“poor world”) or (“poorer countr*”) or (“poorer nation?”) or (“poorer population?”) or (“poorer world”) or (“developing econom*”) or (“less developed econom*”) or (“lesser developed econom*”) or (“under developed econom*”) or (“underdeveloped econom*”) or (“middle income econom*”) or (“low income econom*”) or (“lower income econom*”) or (“low gdp”) or (“low gnp”) or (“low gross domestic”) or (“low gross national”) or (“lower gdp”) or (“lower gnp”) or (“lower gross domestic”) or (“lower gross national”) or lmic or lmics or (“third world”) or (“lami countr*”) or (“transitional countr*”) or (“emerging economies”) or (“emerging nation?”)
